# Hepatomegaly, Elevated Hepatic Enzymes, and Bridging Fibrosis in Patients With Type 1 Diabetes Mellitus

**DOI:** 10.7759/cureus.14446

**Published:** 2021-04-13

**Authors:** Fahad W Ahmed, Tharaga Kirupakaran, Mara Quante, Andrew Smith

**Affiliations:** 1 Diabetes and Endocrinology, University Hospitals Sussex NHS Foundation, Brighton, GBR; 2 Diabetes and Endocrinology, Brighton and Sussex Medical School, Brighton, GBR; 3 Histopathology, University Hospitals Sussex NHS Foundation, Brighton, GBR; 4 Diabetes, Diabetes Care for You and Sussex Community Foundation NHS Trust, Brighton, GBR

**Keywords:** glycogenic hepatopathy, type 1 diabetes, diabetic ketoacidosis, mauriac syndrome

## Abstract

Glycogenic hepatopathy is a rare but reversible condition that includes acute liver dysfunction and hepatomegaly. This occurs due to excessive glycogen accumulation in the hepatocytes. It can occur in patients with poorly controlled type 1 diabetes mellitus. We are reporting a case of a 17-year-old girl who developed liver dysfunction following admission with diabetic ketoacidosis. Ultrasound abdomen confirmed hepatomegaly. However, with improvement in her metabolic control, her liver enzymes and hepatomegaly improved.

## Introduction

Glycogenic hepatopathy (GH) is a clinical condition that occurs due to excessive accumulation of glycogen in hepatocytes. This results in elevated liver enzymes and hepatomegaly [1]. It is a rare and underdiagnosed clinical condition mainly described in type 1 diabetes mellitus (T1DM) [1] Though, it has been reported in type 2 diabetes mellitus (T2DM) as well [2].

The most common DM-related liver complication is a non-alcoholic fatty liver disease (NAFLD). NAFLD is much more common in T2DM [3]. However, a recent meta-analysis has demonstrated that the prevalence of NAFLD is considerable in T1DM [4]. This is likely due to the increased prevalence of obesity in T1DM [4]. It is essential to differentiate between NAFLD and GH as NAFLD is a chronic and progressive condition, whereas GH is an acute but reversible condition.

This case report presents a case of a 17-year-old patient with poorly controlled T1DM who developed hepatomegaly and elevated liver enzymes after the initiation of a high dose of insulin.

## Case presentation

A 17-year-old female with poorly controlled T1DM (HbA1c 130 mmol/mol or 14%) was admitted with diabetic ketoacidosis (DKA). She had a one-day history of vomiting and abdominal pain. On admission, her blood glucose was 20.1 mmol/L.

Examination revealed a body mass index of 21 kg/m^2^, pulse 140 per minute (regular), blood pressure 144/92 mmHg, and her respiratory rate was 21 per minute. Oxygen saturation was 100% on air. Chest, cardiovascular, abdominal, and neurological examination was normal. Investigations are shown in Table 1. Liver enzymes were normal (alanine aminotransferase (ALT): 15 iu/L (0-33) and alkaline phosphatase (ALP): 161 iu/L (20-162).

**Table 1:** Investigation on admission Na: sodium; K: potassium; Hb: hemoglobin; WCC: white cell count; Plt: platelet; CRP: C-reactive protein; BE: base excess

Investigation	Outcome	Normal reference range
Na (mmol/L)	147	135-146
K (mmol/L)	3.5	3.2-5.1
Urea (mmol/L)	7.7	1.7-8.3
Creatinine (µmol/L)	140	44-80
Hb (g/dL)	15.9	11.5-16.5
WCC (x10^9^/L)	20.3	4.0-11.0
Plt (x10^9^/L)	556	150-450
Neutrophil (x10^9^/L)	16.3	2.0-7.5
CRP (mg/L)	<5	<5
pH	7.167	7.35-7.45
PCO_2 _(kPa)	1.43	4.7-6.0
PO_2 _(mmHg)	30.58	82.5-97.5
BE (mmol/L)	-24.8	-2 to +2
HCO_3 _(mmol/L)	3.8	22-29

The patient was started on intravenous insulin as per hospital guidelines. However, following the resolution of DKA, the patient complained of right upper abdominal pain. Abdominal examination revealed an enlarged liver.

**Figure 1:**
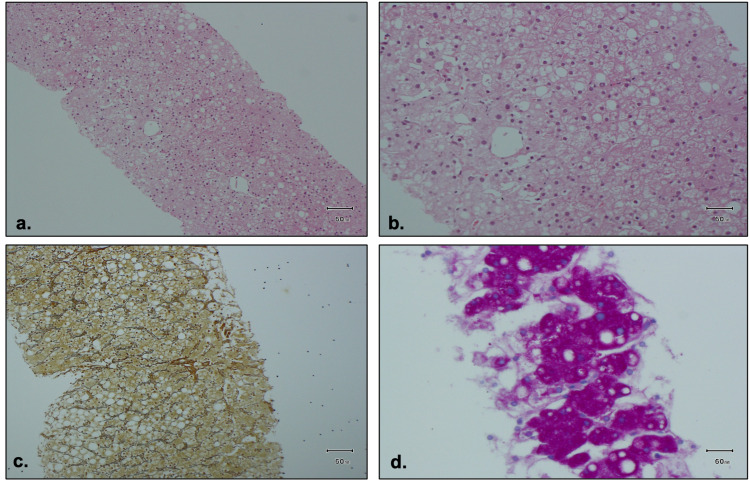
Histology of liver biopsy. (a, b) Hematoxylin and eosin stained shows micro- and macrosteatosis together with ballooning degeneration. (c) Reticulin stained shows bridging fibrosis and paracellular fibrosis. (d) Periodic acid-Schiff stained shows strong positivity in keeping with glycogen

Liver tests showed bilirubin 12 μmol/L (2-17), ALP 213 iu/L, AST 3234 iu/L (0-38), ALT 1097 iu/L, and gamma-glutamyl transferase 295 iu/L (6-42). Ultrasound abdomen showed markedly enlarged liver with diffusely increased reflectivity. All investigations undertaken for abnormal liver function were normal (prothrombin time, international normalized ratio, ferritin, salicylate, paracetamol, immunoglobulin G, A, and M, hepatitis A, B, and C, cytomegalovirus, Epstein Barr virus, copper, ferritin, ceruloplasmin, α-1 anti-trypsin, anti-nuclear, anti-smooth muscle, anti-mitochondrial, anti-gastric parietal, anti-endomysial, and anti-liver-kidney antibody). CT abdomen showed hepatomegaly. She underwent a liver biopsy, which showed macrovesicular steatosis, affecting approximately 50% of hepatocytes. Features of steatohepatitis were not observed. Histochemically abundant glycogen was stained within hepatocytes. Bridging fibrosis was seen on reticulin stain (Figure 1).

**Figure 2:**
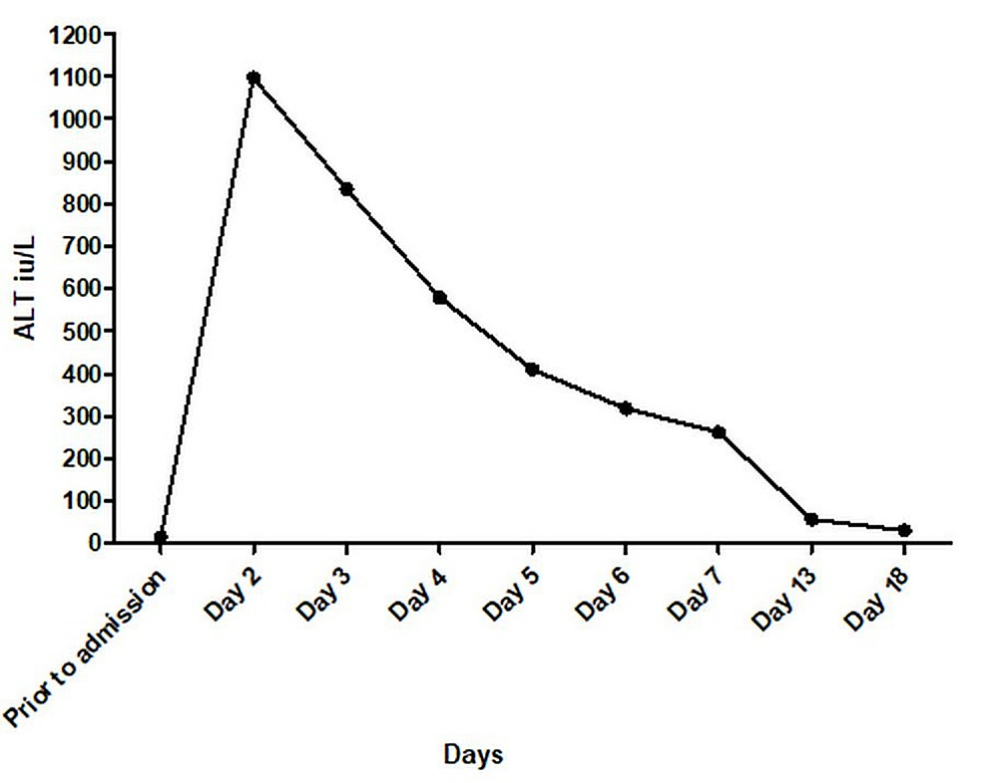
Clinical course of alanine aminotransferase

Her ALT (Figure 2) and AST normalized in 18 days. Hepatomegaly resolved in 13 weeks (repeat ultrasound abdomen showed no organomegaly).

## Discussion

We describe a case of GH in a 17-year-old patient with T1DM. The patient developed acute hepatomegaly and raised liver enzymes following intensive insulin treatment for DKA. However, hepatomegaly and liver enzymes normalized with continued optimal glycemic control.

Mauriac first described transient hepatomegaly and elevated liver enzymes in children with poorly controlled T1DM in 1930 as a part of Mauriac syndrome. Other features included cushingoid facies, dwarfism, delayed sexual maturation, and hyperlipidemia [5]. However, cases of reversible hepatomegaly and elevated liver enzymes were reported without any other characteristics of Mauriac syndrome. This was named glycogen hepatopathy by Torbenson et al. in 2006 [6].

The pathogenesis of GH is unclear. It is proposed that GH occurs due to an imbalance between glycogenesis and glycogenolysis. Glucose usually enters the hepatocytes by passive diffusion through GLUT2, a glucose transporter. This process is insulin-independent [7]. Treatment of hyperglycemia with a high dose of insulin can result in rapid glycogen synthesis in the hepatocytes, resulting in hepatomegaly. This process continues for some time, even after the reduction in the dose of insulin [1].

Almost all of the cases reported have been in T1DM [1]. Few cases have been reported in T2DM [1,2]. Patients usually present with severe hyperglycemia, including DKA. A high dose of Insulin precedes the development of GH. Clinical manifestation of GH ranges from asymptomatic to abdominal pain and jaundice. The physical examination will demonstrate tender hepatomegaly [1]. Rarely, ascites can also be present. This is due to sinusoidal compression due to enlarged glycogen-filled hepatocytes [8]. The biochemical investigation will demonstrate markedly raised AST and ALT by more than 100 times in some cases. It is hypothesized that AST and ALT leak from hepatocyte membranes. ALP can also be raised in some cases. Synthetic liver function is maintained. HbA1c and blood lactate levels are frequently elevated [1].

GH is diagnosed after excluding other common causes of hepatitis (e.g., viral and autoimmune hepatitis, hemochromatosis, Wilson disease, alpha-1 anti-trypsin deficiency, drugs, and rarely glycogenic storage disease). Therefore, it is essential to perform an adequate test to rule out the conditions mentioned above. NAFLD is another important differential diagnosis [1]. 

Radiological investigations (ultrasound, CT, and MRI abdomen) will show an enlarged liver with and without ascites [1]. Ultrasound will not be able to differentiate between GH and NAFLD [1]. The liver will be hyperdense in GH, whereas it will be hypodense in NAFLD on CT scan [9]. Nevertheless, this is not diagnostic [1]. Some studies have demonstrated the utility of dual-echo MRI in differentiating GH from NAFLD [1]. However, further work is needed to establish the sensitivity and specificity of dual-echo MRI to diagnose GH.

Liver biopsy is diagnostic of GH and differentiates it from NAFLD. Histological features on hematoxylin and eosin stain include enlarged pale hepatocytes filled with glycogen. The periodic acid-Schiff stain will demonstrate glycogen stains, which can be digested by the addition of diastase. There is no or minimal inflammation, steatosis, or fibrosis [1]. However, there are reports of cases with minimal fibrosis on histology [10]. This is similar to our patients. The significance of the presence of fibrosis is still unclear and needs further investigation.

GH has a good prognosis and is reversible with improved glycemic control. The liver function and hepatomegaly improve over 2-14 weeks in most cases [1].

## Conclusions

GH remains an underdiagnosed clinical condition with a benign course. It is important to distinguish GH from NAFLD (which is progressive and can lead to liver failure and cirrhosis). This case emphasizes the need for earlier recognition of GH to avoid invasive investigations. Further research is needed to develop a non-invasive test to diagnose GH to avoid a liver biopsy, an invasive test.
